# *In vitro* characterization of the antiviral activity of fucoidan from *Cladosiphon okamuranus* against Newcastle Disease Virus

**DOI:** 10.1186/1743-422X-9-307

**Published:** 2012-12-12

**Authors:** Regina Elizondo-Gonzalez, L Elizabeth Cruz-Suarez, Denis Ricque-Marie, Edgar Mendoza-Gamboa, Cristina Rodriguez-Padilla, Laura M Trejo-Avila

**Affiliations:** 1 Laboratorio de Inmunología y Virología, Facultad de Ciencias Biológicas, Universidad Autónoma de Nuevo León, Ciudad Universitaria, C.P. 66450, San Nicolás de los Garza, Nuevo León, México; 2 Programa Maricultura, Facultad de Ciencias Biológicas, Universidad Autónoma de Nuevo León, Ciudad Universitaria, C.P. 66450, San Nicolás de los Garza, Nuevo León, México

**Keywords:** Fucoidan, NDV, Antiviral, *Cladosiphon okamuranus*

## Abstract

**Background:**

Newcastle Disease Virus (NDV) causes a serious infectious disease in birds that results in severe losses in the worldwide poultry industry. Despite vaccination, NDV outbreaks have increased the necessity of alternative prevention and control measures. Several recent studies focused on antiviral compounds obtained from natural resources. Many extracts from marine organisms have been isolated and tested for pharmacological purposes, and their antiviral activity has been demonstrated *in vitro* and *in vivo*. Fucoidan is a sulfated polysaccharide present in the cell wall matrix of brown algae that has been demonstrated to inhibit certain enveloped viruses with low toxicity. This study evaluated the potential antiviral activity and the mechanism of action of fucoidan from *Cladosiphon okamuranus* against NDV in the Vero cell line.

**Methods:**

The cytotoxicity of fucoidan was determined by the MTT assay. To study its antiviral activity, fusion and plaque-forming unit (PFU) inhibition assays were conducted. The mechanism of action was determined by time of addition, fusion inhibition, and penetration assays. The NDV vaccine strain (La Sota) was used in the fusion inhibition assays. PFU and Western blot experiments were performed using a wild-type lentogenic NDV strain.

**Results:**

Fucoidan exhibited antiviral activity against NDV La Sota, with an obtained IS_50_ >2000. In time of addition studies, we observed viral inhibition in the early stages of infection (0–60 min post-infection). The inhibition of viral penetration experiments with a wild-type NDV strain supported this result, as these experiments demonstrated a 48% decrease in viral infection as well as reduced HN protein expression. Ribavirin, which was used as an antiviral control, exhibited lower antiviral activity than fucoidan and high toxicity at active doses. In the fusion assays, the number of syncytia was significantly reduced (70% inhibition) when fucoidan was added before cleavage of the fusion protein, perhaps indicating a specific interaction between fucoidan and the F0 protein.

**Conclusion:**

The results of this study suggest that fucoidan from *C. okamuranus* represents a potential low-toxicity antiviral compound for the poultry industry, and our findings provide a better understanding of the mode of action of sulfated polysaccharides.

## Background

In the poultry industry, viral infection causes serious losses in productivity with important economic consequences. Antiviral agents are not used because of the high toxicity and elevated production costs associated with the use of most antiviral compounds [1]. Exploring antiviral substances as novel drug candidates is important because of the increasing risks of emerging and reemerging viral infectious diseases. Marine algae contain several metabolites with biological activity that are recognized as promising antiviral agents [2]. The antiviral activity of marine algae polysaccharides against mumps and influenza was reported more than 50 years ago [3].

Fucoidan is a sulfated polysaccharide obtained from marine brown algae that possesses many biological activities including activity against different viruses, such as HIV, herpes simplex virus, dengue virus, and cytomegalovirus [4,5], and exerts significant biological effects on mammalian cells [6]. Its antiviral activity appears to inhibit the initial steps of infection [7]. *Cladosiphon okamuranus* is an edible brown algae that is commercially cultured around Okinawa Island, Japan. Fucoidan is prepared on an industrial scale from algae and used as an additive for health foods, drinks, and cosmetics in Japan. The chemical structure of fucoidan extracted from the brown alga *Cladosiphon okamuranus* was described by Nagaoka et al. According to their findings, fucoidan possesses α 1-3–linked L-fucosyl residues that are substituted with D-glucuronic acid at C-2 and sulfate groups at C-4 of the l-fucosyl residues. The average branched chain structure consists of one sulfate group for every two molecules of fucose and one glucuronic residue for every six molecules of fucose [8]. The polysaccharide also contains xylose as a minor monosaccharide constituent. Using a room temperature extraction, Tako et al. reported an acetylfucoidan yield of 2.3% (w/w) based on the wet alga weight. The total contents of carbohydrates, D-glucuronic acid, sulfuric acid, ash, and moisture were 69, 13.5, 13.6, 23, and 3.2%, respectively, and the molar ratio of L-fucose:D-xylose:D-glucuronic acid:acetic acid:sulfuric acid was estimated to be 4.0:0.03:1.0:2.0:2.0 [9].

Newcastle Disease Virus (NDV) is an enveloped virus that causes severe problems in the poultry industry. It is member of the Paramyxoviridae family, and at least two membrane glycoproteins are responsible for viral entry: the fusion protein (F) and an attachment protein (HN, H, or G, depending on the genus) [10,11]. For NDV, the HN and F proteins are responsible for cell binding and fusion, respectively. The F protein is responsible for fusion of the viral envelope and cell membrane through conformational changes [12]. The F protein is synthesized initially as a precursor, F0, and then cleaved into subunits F1 and F2 by a furin-like enzyme present in the host cell [13]. This cleavage of the F protein is necessary for fusion activation [14]. Cleavage of the F0 protein is a determinant of the pathogenicity and infectivity of NDV [13]. In avirulent NDV strains, the F protein cannot be cleaved by the furin-like enzyme, and it exists as F0. As a result, the fusion process cannot be completed until an exogenous protease such as trypsin is added to facilitate F0 cleavage into an activated form [10]. In our study, we evaluated the anti-NDV activity and mechanism of action of fucoidan from *Cladosiphon okamuranus*.

## Results

### Cytotoxicity of fucoidan and ribavirin

To determine the cytotoxicity of these compounds, we performed MTT assays. No significant cytotoxicity was detected for fucoidan at concentrations up to 100 μg/mL in Vero cells, and a 50% cytotoxic concentration (CC_50_) > 1500 μg/mL was obtained. By contrast, ribavirin exhibited sizeable cell cytotoxicity, with a CC_50_ of 386 μg/mL. The antiviral assays were performed at concentrations below or equal to 100 μg/mL for fucoidan and below or equal to 500 μg/mL for ribavirin.

### Antiviral activity *in vitro*

**Figure 1:**
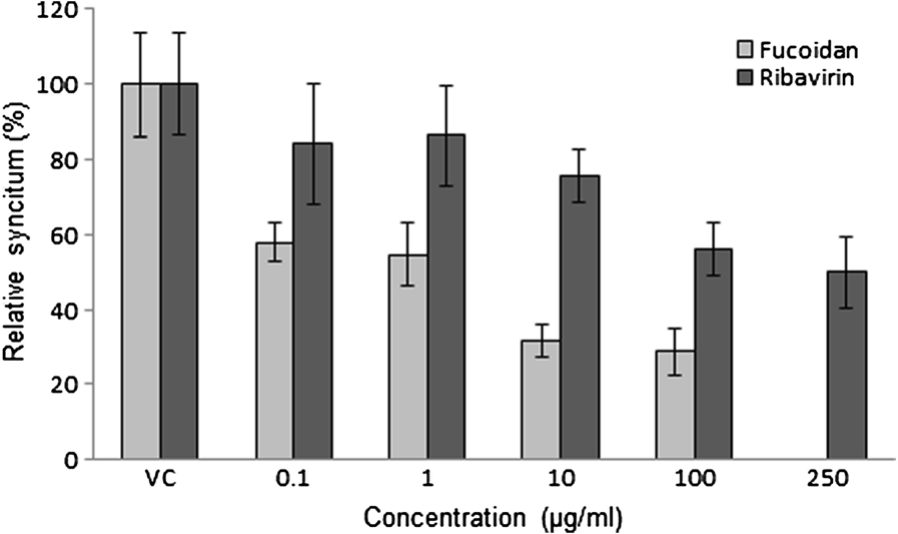
**Antiviral activity of fucoidan against NDV (syncytia reduction).** Vero cells (1.5 × 10^5^ cells) plated in 24-well plates were infected with NDV and treated with different concentrations of fucoidan or ribavirin during and after infection for 48 h, and their antiviral activity was determined in a syncytia reduction assay. The data are expressed as relative syncytia formation (%) to that for the untreated virus-infected control cells, which was defined as 100%. VC, viral control. The data shown are the mean ± standard deviation (SD) from three replicated experiments.

**Figure 2:**
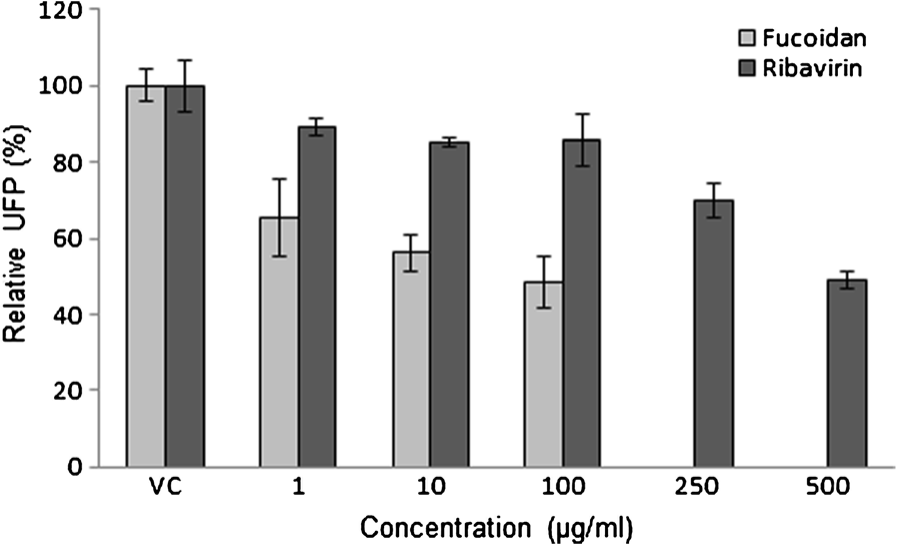
**Antiviral activity of fucoidan against NDV (PFU reduction).** Vero cells (1.5 × 10^5^ cells) plated in 24-well plates were infected with NDV and treated with different concentrations of fucoidan or ribavirin during and after infection for 48 h, and the antiviral activity was determined in a PFU reduction assay. The data are expressed as relative PFU (%) to that for the untreated virus-infected control cells, which was defined as 100%. VC, viral control. The data shown are the mean ± SD from four replicated experiments.

The antiviral activity of fucoidan against NDV was evaluated by syncytia reduction and plaque-forming unit (PFU) inhibition assays. Vero cell monolayers were treated with different concentrations of fucoidan at the time of infection with 800 TCID_50_ or 100 PFUs of NDV, and the concentration of fucoidan was maintained throughout the infection. As shown in Figures 1 and 2, a concentration-dependent inhibition of viral entry into the host cells was observed with the addition of fucoidan compared to the findings in the untreated control cells. The results indicated that fucoidan inhibits syncytia and PFU formation in NDV, with 50% inhibitory concentration (IC_50_) values of 0.75±1.6 μg/mL in the syncytia reduction assay and 58±2 μg/mL in the PFU inhibition assay (mean estimate ± standard error). The selectivity index (SI_50_) of the compound was calculated to be >25.8 for the inhibition of PFU, although the compound displayed a much greater ability to inhibit syncytia formation with a calculated SI_50_ of >2000. We used ribavirin as an antiviral control and obtained IC_50_ values of 224±2 and 490±2 μg/mL in the syncytia reduction and PFU inhibition assays, respectively (SI_50_=0.78 by UFP; Figures 1 and 2).

### Effect of fucoidan on viral infection as determined by time of addition assays

**Figure 3:**
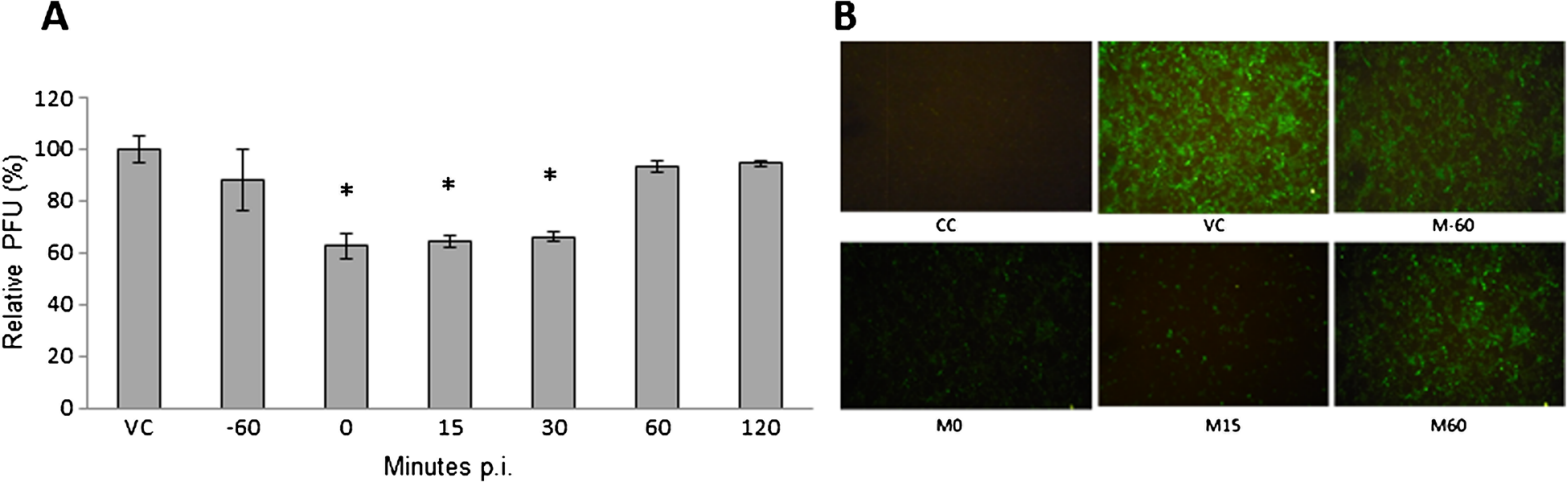
**Time of addition experiments.** Vero cells (3 × 10^5^ cells) plated in 6-well plates were infected with NDV, treated with fucoidan at different times of infection, and analyzed by PFU inhibition assays and immunofluorescence assays. **A**) Fucoidan was added at 60 min preinfection and 0, 15, 30, 60, and 120 min postinfection. The data are expressed as relative PFU (%) compared to that of untreated virus-infected control cells (VC), which was defined as 100%. The data shown are the mean ± SD of triplicate experiments. The asterisks indicate a significant difference between the treatment and viral control (*p<0.05). **B**) Fucoidan was added at 60 min preinfection and 0, 15, and 60 min postinfection with NDV, stained with a mouse monoclonal anti-NDV HN antibody, and analyzed by indirect immunofluorescence as detailed in the Methods.

To determine which step of the NDV cycle was targeted by fucoidan, “time of addition” experiments were performed in Vero cells infected with NDV and exposed to fucoidan at different times of infection. As shown in Figure 3 A, the most efficient inhibition was observed in early phases of infection (0, 15, and 30 min after infection). Fucoidan does not exhibit significant antiviral activity before infection or 1 h after infection. We also confirmed this result in immunofluorescence assays indicating significant inhibition when the compound was added at 0 and 15 min after infection and a weak effect when the compound was added 1 h before or after infection (Figure 3B).

According to the results obtained in the time of addition experiments, the greatest antiviral activity was exhibited before the first hour of infection; however, the compound had no effect when it was added before infection, suggesting that fucoidan inhibits viral penetration into host cells.

### Virucidal activity of fucoidan

To analyze the possibility that this polysaccharide acts directly on the virus particle leading to infectivity inactivation, a virucidal assay against NDV virions was conducted. The virucidal effect of fucoidan against NDV virions was negligible at the maximum tested concentration of 100 μg/mL. This concentration is much higher than the antiviral EC_50_, indicating that the inhibitory effect detected by the plaque reduction assay was actually due to interference with some step of the NDV replication cycle.

### Effect of fucoidan on viral penetration into host cells

**Figure 4:**
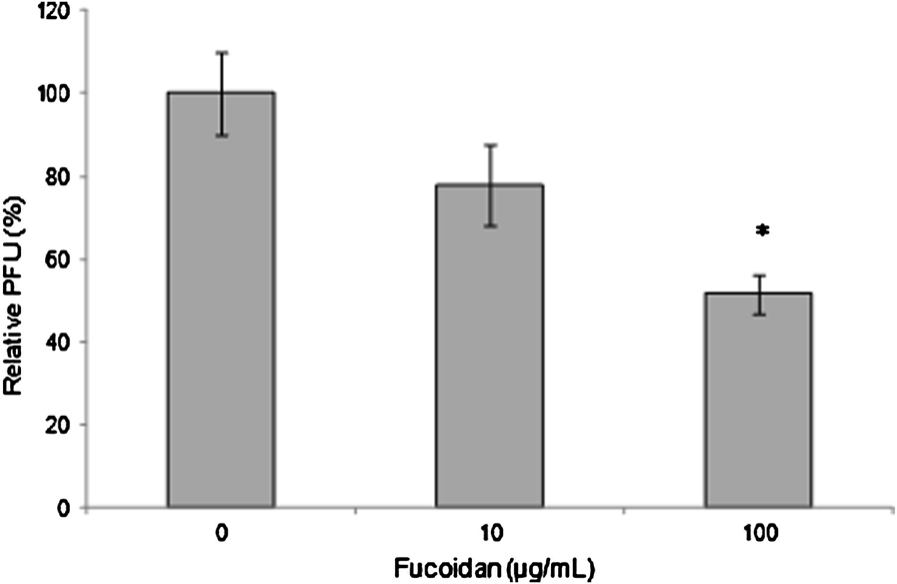
**Effect of fucoidan on viral penetration.** Vero cells monolayers were infected with NDV at 4°C in the absence of fucoidan and then shifted to 37°C to permit penetration of the adsorbed virus in the presence of fucoidan. The effects of fucoidan were evaluated using a PFU inhibition assay. The data shown are the mean ± SD of triplicate experiments. The asterisks indicate a significant difference between the treatment and viral control (*p<0.05).

To determine whether entry events downstream of virus binding were inhibited by fucoidan, Vero cells were incubated with NDV at 4°C for 1 h to allow virus binding but prevent viral internalization. Unbound virus was washed away, prewarmed medium containing 0, 10, or 100 μg/mL fucoidan was added to the cells, and the cells were then shifted to 37°C for the remainder of the experiment. In these experiments, 10 μg/mL fucoidan significantly decreased viral infection by 23% and 100 μg/mL fucoidan significantly decreased viral infection by 48% compared with the findings in infected cells in the absence of treatment (Figure 4). These findings supported the possibility that postbinding events are inhibited by fucoidan.

### Effect of fucoidan on NDV protein synthesis

**Figure 5:**
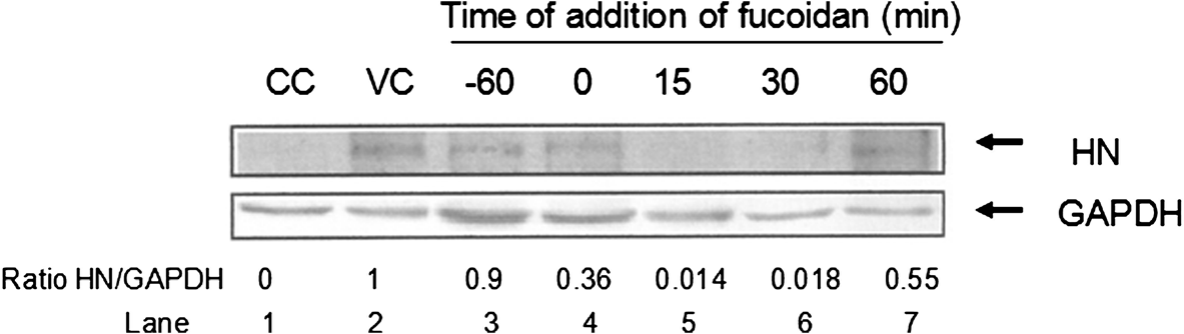
**NDV HN protein levels in Vero cells infected with NDV and treated with fucoidan.** Vero cells (3 × 10^5^) were infected with NDV and treated with 100 μg/mL fucoidan at different times of infection. Lane 1, cellular control without NDV infection (CC); Lane 2, viral control without fucoidan treatment (VC), Lanes 3–7, cells infected with NDV and treated with fucoidan at 60 min before and 0, 15, 30, and 60 min after infection, respectively. Cells lysates were prepared, and equal amounts of protein extracts (40 μg) were subjected to immunoblot analysis to detect HN and GAPDH levels. The ratio of HN/GAPDH expression in the immunoblot analysis was quantified using Phoretix1D v2003.02 software.

Considering the results, we decided to evaluate the effect of fucoidan by measuring the relative concentrations of viral protein synthesized in the cells exposed to the compound at various stages of infection. When fucoidan was added to the Vero cell monolayers at the time of viral infection, HN protein levels were decreased by 64%; however, when the compound was added 15 or 30 min after infection, HN protein levels were dramatically decreased by 98.6 and 98.2%, respectively. The compound had a lower effect when added 60 min after infection (Figure 5).

These results are consistent with those observed in trials of PFU and support the possibility that postbinding events are likely responsible for the inhibition of NDV infectivity by fucoidan.

### Ability of fucoidan to block NDV-induced cell-cell fusion

**Figure 6:**
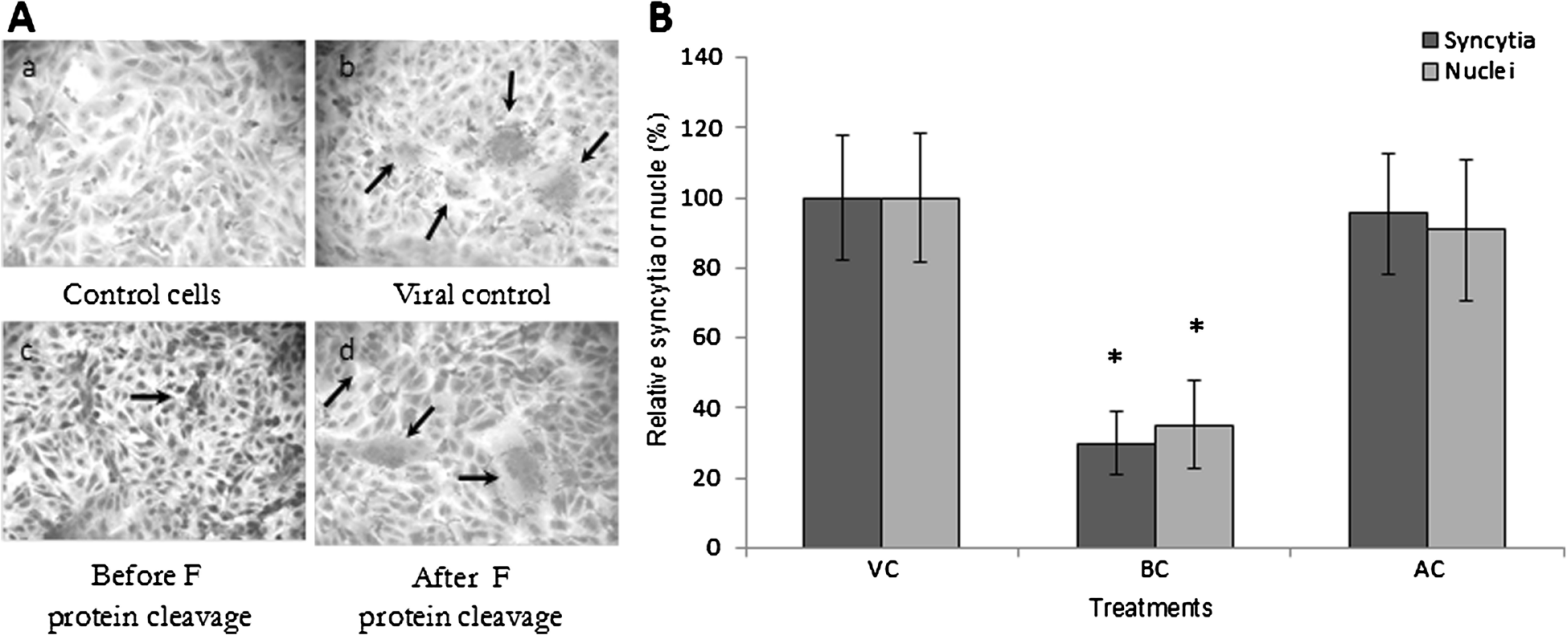
**Inhibition of avirulent NDV (La Sota) strain-mediated cell fusion. A**) Vero cells infected with NDV and treated by trypsin digestion: a) Uninfected control cells; b) Vero cells infected with NDV and treated with trypsin; c) Infected Vero cells were treated with fucoidan before F protein cleavage, at which point syncytia formation could be inhibited if fucoidan was added; d) Infected Vero cells were treated with fucoidan after F protein cleavage, and fucoidan could not inhibit syncytia formation in this condition. **B**) Fusion inhibition assay with fucoidan treatment before (BC) and after F protein cleavage (AC). Data are expressed as percent of the number of syncytia and syncytial nuclei compared with the findings in untreated virus-infected control cells. VC, viral control. The data shown are the mean ± SD of triplicate experiments. The asterisks indicate a significant difference between the treatment and viral control (*p<0.05).

To determine whether the compound inhibits the cell-cell spread of NDV, we performed cell fusion inhibition assays as described in the Methods. Avirulent strains of NDV are characterized by their inability to form syncytia because the F protein cannot be cleaved. However, after trypsin digestion, syncytia formation is observed. The results indicated that fucoidan inhibited syncytia formation only if it was added before F protein cleavage (before trypsin digestion; Figure 6A). Before F protein cleavage, fucoidan inhibited syncytia formation by 70% and decreased the number of syncytial nuclei by 64.8% compared with the findings in untreated viral-infected control cells. However, after F protein cleavage via trypsin digestion, fucoidan lost the ability to inhibit syncytia formation (Figure 6B). This suggests that fucoidan inhibits viral fusion by interacting with the intact F0 protein but not with the mature F protein (i.e., the activated form of the F_1_/F_2_ complex).

## Discussion

Newcastle disease (ND) is one of the most serious infectious diseases affecting birds, particularly poultry, and it has been the cause of serious economic losses [1]. There is no treatment for ND. Vaccination is practiced widely, and it remains the recommended method for prevention. Although vaccination using live and killed vaccines is widely used as a management practice, the velogenic strains are endemic in the commercial poultry of many countries [15,16]. This represents a major problem for the poultry industry and therefore requires further measures to prevent and control this disease.

Antiviral treatments are not available in poultry due to their cost and toxicity, and thus, a promising alternative is the use of compounds of natural origin, which have been underexplored for this purpose [17,18]. Several sulfated seaweed polysaccharides have been demonstrated to exhibit antiviral activity against a wide spectrum of viruses [4,5,7,19] with effectiveness that depends on the sugar composition, main chain length, sulfation level, and sulfate pattern [20,21]. An advantage of sulfated polysaccharides from algae is the high content of polyanions in their extracellular matrix; consequently, they can be prepared and made available at a very low cost, and sulfated polysaccharides isolated from many marine algae possess species-specific structural variations that appear to affect their antiviral activity [22].

The major undesirable side effect of sulfated polysaccharides is their well-known anticoagulant activity. This adverse effect can be avoided by selecting sulfated polymers, such as fucoidan from *C. okamuranus*, which exhibit virtually no anticoagulant activity [23]. Cumashi et al. suggested that the high presence of glucuronic acid branches is the most likely feature responsible for the lack of anticoagulant activity by *C. okamuranus* fucoidan, as the less active compounds are characterized by a low degree of sulfation and a high presence of 2-*O*-α-D-glucuronyl substituents along the linear polysaccharide backbone (Characteristics fucoidan from *C. okamuranus*) [23]. By contrast, it has been proposed that the antiviral activity of fucoidan is related to the concentration of fucose and uronic acids. Hidari et al. found that the antiviral properties of fucoidan from *C. okamuranus* against dengue virus type 2 vanished when the glucuronic acid was carboxyl-reduced [5], and Jiao et al. indicated that a carboxyl-reduced fucoidan derivative from *C. okamuranus*, which contained fucose and sulfate groups but no uronic acids, did not display significant antiviral effects against influenza A [21]. In the present study, we evaluated the antiviral activity of fucoidan from *C. okamuranus* extracted by the method of Tako et al. with a significant degree of uronic acids, and investigated its possible mechanism of action in Vero cells [9].

There are very few drugs available for the treatment of infections caused by RNA viruses. Ribavirin inhibits many paramyxoviruses *in vitro*, such as parainfluenza, the measles virus, the mumps virus, RSV, and canine distemper virus [24,25]. Ribavirin is approved for the treatment of RSV infection in children; however, the efficacy of ribavirin against RSV is limited [26]. In our work, ribavirin did not display good antiviral activity because its IC_50_ was 490, and its CC_50_ was close to that value (SI_50_=0.78). Conversely, according the findings, no significant *in vitro* toxicity was observed with fucoidan*,* as its CC_50_ was *>*1500 μg/mL. Previous studies reported low *in vitro* cytotoxicity for sulfated polysaccharides from different algal sources, consistent with our findings for fucoidan [27]. Additionally, in the study by Gideon and Rengasamy, rats treated with *C. okamuranus* exhibited no necropsy or other pathological changes in organs or changes in histopathological morphology, consistent with the lower toxicity possessed by sulfated polysaccharides, specifically fucoidan from *C. okamuranus*[28].

Fucoidan from *C. Okamuranus* exhibited good antiviral activity against NDV, with an SI_50_ of >25.8, although the compound displayed a much greater ability to inhibit syncytia formation, with a calculated SI_50_ of >2000. Therefore, fucoidan from *C. okamuranus* much more effectively inhibited NDV. Similar results were obtained for other sulfated polysaccharides from different algae against several enveloped viruses [5,19,26,29]; however; it appears clear that specific interactions between viruses and sulfated polysaccharides are related to the particular characteristics of the viruses and compounds. Variations in the amino acid sequence of the viral envelope glycoproteins result in differential susceptibilities to the compounds that interact with them [30-33]. Studies of structures related with the antiviral effect of *Cladosiphon* fucoidan against dengue viruses strongly suggested that both glucuronic acid and sulfated fucose residues in fucoidan appear to critically affect its antiviral effect [5]. Fucoidan from *C. okamuranus* exhibited significant antiviral activity against NDV infection in this study, therefore suggesting a specific inhibition of NDV infection in host cells.

We found a virucidal effect negligible of fucoidan against NDV virions, indicating that the inhibitory effects detected by the inhibition syncytia or plaque reduction assays were actually due to interference with some step of the NDV replication cycle. The lack of virucidal activity for fucoidan from *C. okamuranus* is in accordance with previous studies that found most algal sulfated polysaccharides cannot induce significant virion inactivation [34-36].

According our results obtained by time of addition assays, fucoidan exerted an inhibitory effect on the early phases of the virus-cell interaction (Figure 3). These results agree with previously reported data indicating that algae-derived sulfated polysaccharides can inhibit viral infection by interfering with the binding and penetration of the virus into cells [5,37]. We further confirmed the antiviral activity by Western blotting using NDV-infected Vero cells that were treated with fucoidan at different times after infection. Our results clearly reveal marked inhibition of HN protein expression, with the greatest inhibition obtained at 15 min post-infection (Figure 5). These results support the ability of fucoidan to block the early stages of infection.

As judged by our findings, fucoidan acts in early steps of infection and inhibits syncytia formation. Therefore, we decided to determine the specific viral step(s) inhibited by fucoidan. The NDV envelope contains two proteins related to entry: the attachment protein HN and the fusion protein F. Fusion of the viral envelope with the cell membrane occurs after attachment via conformational changes in the F protein that are specifically arrested at 4°C. The addition of fucoidan after prebinding of NDV to cells effectively blocked viral infection (Figure 4). These findings suggest that fucoidan principally inhibits NDV infectivity by blocking one or more postbinding entry steps.

As described in this study, although the primary antiviral targets of fucoidan were suggested to be early stages of NDV infection, fucoidan inhibited the cell-to-cell spread of NDV when added to the medium at 12 h postinfection. Regarding avirulent NDV strains, fusion did not occur without the addition of trypsin in the fusion inhibition assays with NDV La Sota, and we only observed antiviral activity when fucoidan was added before F protein cleavage, indicating that the compound inhibits fusion, perhaps via a direct effect on the F0 protein (Figure 6). As a consequence of this mode of action, fucoidan both inhibits the penetration of NDV to cells and strongly suppresses NDV-induced syncytia formation between NDV-infected cells and uninfected cells, a process that drastically enhances NDV spread and infectivity. Our results agree with those of Parskaleva et al., who demonstrated the ability of an extract from *Sargassum fusiforme* to inhibit the entry events of HIV and the mechanism by which HIV infection spreads [38].

In the penetration inhibition assays, we observed a reduction in viral infection when fucoidan was added after adsorption. As the cellular receptor to which the virus binds is already known (Figure 4), this finding suggests that the inhibitory effect of fucoidan on the viral F protein may be principally responsible for this inhibition and not an effect on the cellular receptor (when the F protein is possibly exposed to the compounds). However, we cannot rule out an effect on the HN protein, as the fusion mediated by the NDV F protein absolutely requires the participation of the HN protein [12]. Our findings suggest that the inhibition of virus fusion events (virus-cell and cell-cell) may be principally responsible for the inhibition of NDV infectivity by fucoidan from *C. okamuranus*.

## Conclusion

Fucoidan inhibits NDV *in vitro*, and it did not exhibit significant toxicity at effective concentrations. Fucoidan exerted no direct inactivating effect on virions in a virucidal assay. Our results support the concept that fucoidan acts in early stages of viral infection so as to inhibit viral-induced syncytia formation, probably by blocking the F protein. It might be concluded that fucoidan represents a promising antiviral for the poultry industry that may prevent NDV infection.

## Methods

### Antiviral agents

Fucoidan was purchased as a dried powder from Kadoya & Co., Kobe, Japan (lot A03012), extracted (as described by Tako et al., 2000) from cultured kelp *Cladosiphon okamuranus* harvested off the coast of Okinawa Island, Japan. The polysaccharide preparation was certified to contain 90.4% fucoidan (anthrone-sulfuric acid method) and exhibit a mean molecular weight of 92.1 kDa (HPLC method). The fucose and sulfate contents of fucoidan were of 38.6 and 15.9%, respectively, with ash comprising 19.6% of the content and other sugars comprising 23% (glucuronic acid and traces of xylose). Dried samples of fucoidan were suspended in Dulbecco’s modified Eagle’s medium (DMEM) at a concentration of 2.5 mg/mL and filtered through a membrane filter (pore size, 0.22 mm). Ribavirin (Vilonapediatrica, Valeant, México) was used as an antiviral control.

### Cell lines and viruses

Green African monkey kidney (Vero) cells and chicken embryo fibroblasts were grown in DMEM/F-12 supplemented with 10% (v/v) fetal bovine serum and 1% (v/v) antibiotics. The flasks were maintained in a humidified atmosphere with 5% CO_2_ at 37°C. The La Sota and wild-type lentogenic NDV strains were propagated in 9-day-old chicken embryo eggs. Stock viruses were harvested, titrated, and stored at −70°C until used. Both strains were titered by hemadsorption and according to their PFUs.

### Cytotoxicity assay

The cytotoxicity of the compounds was evaluated by MTT reduction assays. Vero cells were seeded in 96-well plates at an initial density of 5 × 10^3^ cells per well. The cells were incubated with increasing concentrations of the compounds for 48 h at 37°C and 5% CO_2_. MTT solution (5 mg/mL) was added to the cells, which were further incubated for 4 h. MTT was removed, and 100 μL of DMSO were added for 5 min. The optical density was measured at 570 nm (Microplate Autoreader EL311; BIOTEK Instruments Inc. USA). Each experiment was performed in sextuplicate, and experiments were repeated at least three times. The cytotoxicity was expressed as the CC_50_, which was the concentration of the test substances that inhibited the growth of Vero cells by 50% compared with the growth of the untreated cells.

### Antiviral activity

The antiviral activity of fucoidan against NDV was assessed using syncytia formation or plaque reduction assays with monolayers of Vero cells grown in 24- or 6-well plates. The assays were performed adding the compounds during all infection cycles*.* Generally, 100 PFUs or 800 TCID_50_ of NDV were incubated with different concentrations of drugs for 1 h at room temperature. The virus was allowed to adsorb onto the cells for 1 h at 37°C. The residual inoculum was discarded, and cells were washed three times with PBS, after which DMEM (for syncytia assays) or medium containing 0.6% agar and 0.001% trypsin (GIBCO; for PFU assays) was added to the cells. Each concentration was investigated using three culture wells per fucoidan concentration per experiment, and the experiments were repeated three and four times for the syncytia and plaque formation assays, respectively; ribavirin was used as a control. Monolayers were fixed with methanol:acetone after incubation for 48 or 72 h at 37°C in a 5% CO_2_ incubator and stained with 1% crystal violet, and subsequently, syncytia or plaques were counted. By reference to the number of syncytia or plaques observed in viral control monolayers (untreated cultures), the IC_50_ was determined from dose–response curves.

### Virucidal assay

The virucidal activity of fucoidan against NDV was assessed using plaque reduction assays with monolayers of Vero cells grown in 6-well plates. The assays were performed by adding the compound (0, 10, or 100 μg/mL) to an equal volume to NDV (100 PFU/100 μL). After 0, 1, 3, or 6 h, the mixtures were added to Vero cells for 1 h at room temperature. Thereafter, the cells were washed three times with PBS, and medium containing 0.6% agar and 0.001% trypsin (PFU assays) was added. Monolayers were fixed with methanol:acetone after incubation for 72 h at 37°C and 5% CO_2_ and stained with 1% crystal violet in an incubator, after which plaques were counted.

### Time of addition assay

Vero cell monolayers were infected with 800 TCID_50_ or 100 PFUs of NDV. Fucoidan was added at a concentration of 100 μg/mL at different times of infection: 60 min preinfection, 0, 15, 30, 60, and 120 min postinfection (Figure 4A), and 3, 5, and 7 h post-infection (data not shown). Thereafter, for each treatment, cells were incubated with fucoidan for 1 h and then washed three times with PBS, and medium containing 0.6% agar and 0.001% trypsin (PFU assays) was added. Monolayers were fixed with methanol:acetone after incubation for 48 or 72 h at 37°C and 5% CO_2_ and stained with 1% crystal violet; subsequently, plaques were counted.

### Immunofluorescence

Vero cells grown on glass coverslips were treated with 100 μg/mL fucoidan at different times of infection (60 min before infection and 0, 15, and 60 min postinfection) with 100 PFU/mL NDV. After 1 h at 37°C, the cells were washed three times with PBS, and 12 h later, the monolayers were fixed with 1:1 methanol:acetone. Thereafter, the cells were washed three more times with PBS, incubated with blocking solution (PBS containing 1% BSA (w/v) for 30 min, and then incubated 2 h with Newcastle monoclonal anti-HN antibody (dilution 1:100, Chemicon International, CA). After washing three times with PBS/Triton, the cells were incubated with bovine antimouse IgG-FITC (dilution 1:100, Santa Cruz). After washing three times with PBS/Triton, the slides were mounted with Vectashield medium and visualized using a confocal laser-scanning microscope (Olympus IX70, USA).

### Viral penetration assay

Virus penetration into Vero cells was evaluated according to the method reported by Huang and Wagner [29] and modified by Highlander *et al.*[30]. Vero cell monolayers precooled at 4°C for 3 h were infected with 100 PFUs of NDV at 4°C for 1 h in the absence of fucoidan. After washing three times with ice-cold PBS, different concentrations of fucoidan were added to the monolayers, and the temperature was shifted to 37°C. After 30 min or 1 h of incubation at 37°C, the cells were treated with 40 mM citrate buffer (pH 3.0) to inactivate unpenetrated viruses. The cell monolayers were overlaid with 0.6% agar in DMEM and trypsin and subjected to PFU assays.

### Protein extraction and immunoblot assay

Total cell lysates were prepared from Vero cells infected with NDV and treated with fucoidan at different times of infection. Proteins were extracted with 1× lysis buffer containing 10 mM Tris–HCl pH 7.5, 50 mM KCl, 2 mM MgCl_2_, 1% Triton X-100, 1 mM dithiothreitol, 1 mM phenylmethylsulfonyl fluoride, and complete EDTA-free protease inhibitors. After incubation on ice for 20 min, the lysates were centrifuged at 13,000 × *g* for 10 min at 4°C.The amount of protein in the supernatant was measured by the Bradford assay (Bio-Rad). Equal amounts of protein (40 μg) were separated by 10% SDS-PAGE and transferred onto nitrocellulose membranes (Hybond-ECL Amersham Biosciences). The membranes were blocked with phosphate-buffered saline (PBS)/Triton (0.1% Triton X-100 in PBS, pH 7.2) supplemented with 5% of BSA (w/v) for 1 h and then incubated overnight at 4°C with Newcastle monoclonal anti-HN antibody (dilution 1:500, Chemicon International CA) or mouse monoclonal anti-GAPDH antibody (dilution 1:500, Chemicon International CA). After washing with PBS/Triton, the membranes were incubated with HRP-Goat Anti-Mouse IgG Conjugate (dilution 1:1000, Chemicon International, CA, USA) for 2 h at room temperature. Peroxidase-coupled antibodies were detected using an enhanced chemiluminescence detection system (Roche).

### Fusion inhibition assay

Fusion inhibition assays were performed according to the method reported by Zhu Jieqing [10]. Vero cell monolayers in 24-well plates were infected with 800 TCID_50_ of the avirulent NDV strain (La Sota). After 12 h of infection, the monolayers were washed three times with PBS, digested by trypsin (GIBCO) in DMEM at room temperature for 20 min, and then washed three times with PBS, and the monolayers were incubated in DMEM containing 5% fetal bovine serum for 12 h. Monolayers were fixed with 1:1 methanol:acetone and stained with 1% crystal violet. To detect its ability to inhibit fusion, fucoidan was added to the medium before or after trypsin digestion.

### Statistical analysis

The data were analyzed by SPSS 15 software. All variables were tested in triplicate for each experiment, and experiments were repeated at least three times. The CC_50_ and IC_50_ values were determined by probit regression analysis. One-way analysis of variance was performed followed by Dunnett’s test. Values of *p<0.05 were considered statistically significant.

## Competing interests

The authors declare that they have no competing interests.

## Authors’ contributions

Conceived and designed the experiments: LTA, REG. Performed experiments: REG. Analyzed the data: REG, LTA, ECS, DRM, CRP. Wrote the paper: REG, LTA, EMG. All authors read and approved the final manuscript.
